# Coordination strategies and concept of operations implemented during activation of public health emergency operations center for COVID-19 response in Pakistan

**DOI:** 10.1038/s41598-023-46234-5

**Published:** 2023-11-01

**Authors:** Majid Ali Tahir, Mumtaz Ali Khan, Aamer Ikram, Tamoor Hamid Chaudhry, Afreenish Amir, Muhammad Tahir, Ijaz Ul Haq, Shahbaz Ahmed Zaki, Arslan Salam, Sidra Wali, Wasay Munir, Muhammad Salman

**Affiliations:** 1 CDC, National Institutes of Health, Islamabad, Pakistan; 2 Department of Public Health and Nutrition, University of Haripur, Haripur, KP Pakistan; 3 Center for Disease Control, National Institutes of Health, Islamabad, Pakistan; 4 Federal Medical College, Islamabad, Pakistan; 5 University of Lahore, Lahore, Pakistan

**Keywords:** Health care, Health occupations, Medical research

## Abstract

Public health emergency management systems encountered difficulties in developing countries, especially in Pakistan. The COVID-19 pandemic was extremely challenging for different agencies/departments in Pakistan. Health emergency management depends on a well-established public health emergency operations center that could generate a coordinated response to emergencies. We conducted an assessment of public health emergency response coordination implemented during the COVID-19 at strategic level. This was mix-method qualitative study. Primary data was collected by using a structured questionnaire, and secondary data was collected by desk review. The agencies engaged in pandemic response at the national level in Pakistan were included in the assessment. The overall score of the emergency response coordination system during COVID-19 was 49% for all agencies. We found that agencies faced challenges in leadership, legislation, and financing issues during the pandemic response (44%). None of the agencies had a fully developed framework for joint planning and response system for health emergencies. Roles and responsibilities attached to designated agencies in response were relatively clear (55%) for most of the agencies. Effective public health emergency response is based on multi-departmental coordination, resource mobilization, and clear roles for each agency. Pakistan must proactively address these challenges for pandemic response in future.

## Introduction

Mass travel and trade make it challenging for the world to prevent and control infectious diseases. Increased movement of people, containers, and commodities allows disease pathogens to spread worldwide within a very short time span of time. The geographical spread of COVID-19 has brought many challenges for world populations since January 2020^1^. The emergence of SARS-CoV-2, the seventh coronavirus known to be transmitted in human from animals, quickly led to the global challenge of the COVID-19 pandemic^2^. Despite prior planning and preparedness exercises conducted by many countries, its rapid spread, caught public health systems off guard^1,3^. The world’s healthcare systems lacked the necessary knowledge to effectively combat this new pathogen. Most of the countries began focusing on enhancing disease detection, information coordination, resource allocation, legal support, and dedicated funding to address the crisis^4^.

In 2015, WHO urged Member States to develop capacity in Incident Management Systems (IMS) and to establish a public health emergency operations center (PHEOC) to manage public health emergencies more effectively^2,5,6^. In response to COVID-19, most counties activated their PHEOCs, which have been considered the most efficient systems for response^7^. Emergency management system in PHEOC framework is built on eight pillars including, legal authority, steering committee, objectives of operations, essential functions, its core components, training & exercises, Monitoring & evaluation and sustained financing^5^. Planning includes emergency response plan and Concept of Operations (CONOPs). A concept of operations is defined as the intended operation of emergency response system including structure, levels of response, components of response and plan that guides how to engage different branches and levels of government as well as other partners in emergency response^5,7^.

Government of Pakistan has established PHEOC at National Institutes of Health (NIH) Islamabad, with support of CDC United States in 2018^8^. Before PHEOC, Pakistan had a functional National EOC (NEOC) responding to endemic Polio. PHEOC was first time activated for dengue response with limited capacity in 2019^9^. In 2020, recognizing the emerging threat of COVID-19, the PHEOC was once again activated to coordinate information at the national level^10^. The newly emerged situation confronted the whole public health system at strategic, operational, and tactical levels.

The healthcare system of Pakistan is composed of federal and provincial autonomous administrations with dedicated resources and legal status for preparedness and response. The federal level institutions oversee national health policies and initiatives, while individual provincial health departments manage and administer healthcare services at the provincial level with their own resources^11^. Due to the devolving nature of the health system in Pakistan, every province has its own public health emergency management system. Similarly, most operational and tactical preparedness activities were carried out by the provinces in coordination with federal government^12^.

As the pandemic evolved and given the scale of emergency, the Government of Pakistan established a new structure of incident management system with name of National Command & Operation Centre (NCOC) 27th March 2020^13^. The new structure functioned for two years. In April 2022, the NCOC was redesignated and PHEOC at NIH was officially named as NCOC by with executive directives in 2022^14^. This emergency response coordination system was linked with various stakeholders across the country, necessitating its review during and after activation. The system’s performance and effectiveness are evaluated through the continuous assessment and review of its core functions.

This study aimed to explore how the incident management system in Pakistan developed coordination mechanism for pandemic response at strategic level in Pakistan. The study designed to highlight existing relationships and concept of operations at the strategic level in response to COVID-19 pandemic in Pakistan. This study provided technical understanding about some essential functions of public health emergency operations center (PHEOC) in developing effective response to the public health emergencies.

## Methods

### Study design

This was a mixed method qualitative study. A combination of primary and secondary data collection was used to review and analyze the emergency response coordination system during COVID-19 pandemic in Pakistan. Data was collected through structured questionnaire from the respondents. Moreover, a desk review analysis was performed to analyze the secondary data related to organizations and agencies involved in pandemic response.

### Study settings

This study assessed the coordination mechanism of different agencies and developmental partners working for COVID-19 response in Pakistan at federal level. Data were collected from 18 federal-level agencies/departments/sections and developmental partners, identified in the National Action Plan for COVID-19, Pakistan^10^. These agencies/departments were assigned different tasks during the COVID-19 pandemic preparedness, support response operations and risk mitigation. The involved agencies and partners were 1-National Command and Operations Center (NCOC), 2-National Disaster Management Authority (NDMA), 3-National Health Emergency Preparedness & Response Network (NHEPRN), 4-National Emergency Operations Center (NEOC), 5-Health Services Academy (HSA), 6-Ministry of Interior (MOI), 7-Ministry of Defense (MOD), 8-Directorate of Programs, 9-Ministry of Religious Affairs (MORA), 10-Ministry of Industries and Production (MOIP), 11-Ministry of Information and Broadcast (MOIB), 12-Federal Directorate of Immunization (FDI), 13-Boarder Health Security (BHS), 14-Health Research Institute (HRI), 15-District Health Department (DHO) Islamabad, 16-WHO Pakistan Office, 17-Jhon Snow Research Institute (JSI) Pakistan Office and 18-Health Security Agency UK (UKHSA) Pakistan Office.

### Selection criteria

Theses eighteen agencies and partners were included in National Action Plan (NAP) and assigned different tasks from NCOC time to time during COVID-19 response. These agencies operate independently, although sometimes they coordinate in order to facilitate public health preparedness and response for national emergencies with specialized roles and responsibilities.

### Study duration

This study was conducted from October 2022 to January 2023.

### Study tool

A self-administrated structured data collection tool was developed in the light of “WHO Toolkit for Assessing Health-System Capacity for Crisis Management”^15^, and WHO’s framework for PHEOC-2015^5^. It consisted of four indicators, including (a) leadership, legislation and financing, (b) concept of operations, (c) coordination and collaborations, and (d) human resources and workforce. Statements in the tool was customized and tailored according to the objectives of the study and with reference to Centers for Disease Control (CDC) guidelines for Public Health Emergency Preparedness and Response Capabilities^16^.

### Data collection

Data were collected from subject matter experts of the respondent agencies and their sections. We arranged the meeting with informants for interview to collect data. Data collection tool contained multiple question in each indicator. Face to face interviews were conducted for compellation of the questionnaire. The secondary data was also obtained, including supporting documentation, official information, and evidence of coordination and response. Primary data was collected from 18 agencies, including 15 public sector organizations or departments and 3 developmental partners. From all the agencies, total of 47 subject matter experts were nominated as interviewees based on their area of expertise. A detailed desk review of relevant documents was performed along with primary data analysis. An informed consent was obtained from all of the respondents of the study who were interviewed.

### Data analysis

Descriptive statistics were calculated by assigning values for the qualitative attributes including Yes = 100%, Partial = 50%, and No = 0%^17^. All the methods of data collection and analysis were carried out in accordance with the WHO’s guidelines and regulations for Core Capacity Requirements Assessment Tool for PoE-2009^17^. Levels of indicators in the study expressed in percentages. One indicator consisting of multiple questions. The average of each indicator was calculated for all individual agencies. Finally, cumulative percentage of their average scores were calculated from public health emergency planning, preparedness, and initial response indicators^17^. For each agency the average of multiple responses were taken as one response. Cross-tabulation was also used to demonstrate agency wise roles in pandemic response.

### Ethical approval and informed consent

This is a system level assessment study focused on the roles of different agencies in emergency response. Although the study did not involve human as subject of the study, however an IRB approval also obtained from the Institutional Reviewed Board, of National Institutes of Health, Islamabad Pakistan.

## Results

**Figure 1:**
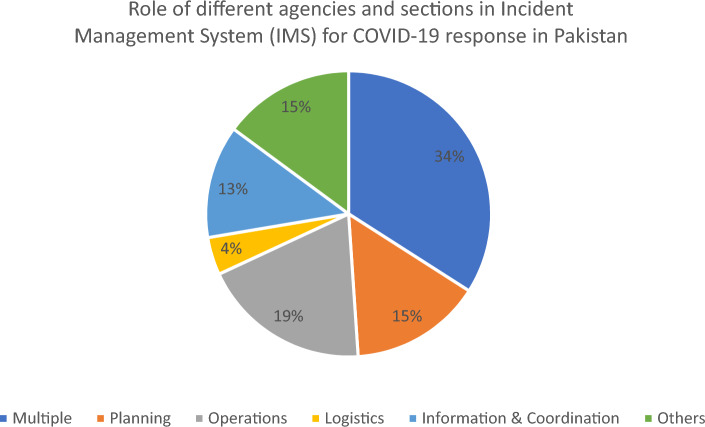
Role of different agencies and sections in Incident Management System (IMS) for COVID-19 response in Pakistan.

Leadership, legislation, and financing were found to be major issues for the agencies engaged in pandemic response. Most of the responses (n = 44) were collected from the public sector organization, department, or the sections. Majority (n = 32) have primary role in the healthcare management system at national level *(*Table 1*)*. Among all organizations majority (34%) claimed that they have multiple roles during COVID-19 response including liaison, communication, planning, operations, and logistics etc. (Fig. 1). While the other were not clear about their specific roles, as they were called occasionally for some services.

**Table 1:** Type of organizations & agencies of the study.

Variables	Attributes	Frequency (%)
Respondent’s agencies and sections	Public sector	44 (94%)
	Development sector	3 (7%)
Primary function of the agencies	Health	32 (68%)
	Non-health sector	15 (32%)

The overall average for all four indicators was 49% of all agencies (Table 2). Most of the respondents claimed they their agencies has a partial legislative support for national planning and response to COVID-19 pandemic. Out of health sector agencies, 41% claimed that their agency has membership in high- level multisectoral committee at national level during COVID-19 response (Table 3)*.* Lack of coordination was observed in joint planning and response activities. Regarding trained workforce, the agencies in health sector do not have a maintained database of staff trained in emergency response and coordination.

**Table 2:** Agency wise scores for each Indicator during Emergency Response for COVID-19.

Agencies and partners	Leadership, legislation and financing (%)	Concept of operations in COVID-19 response	Coordination and collaborations (%)	Resources and workforce for response and coordination (%)	Average in all indicators for the agencies (%)
Agency 1	75	83	75	67	**75**
Agency 2	25	50	25	17	**29**
Agency 3	25	58	42	50	**44**
Agency 4	67	92	92	92	**86**
Agency 5	17	33	33	25	**27**
Agency 6	8	33	17	25	**21**
Agency 7	25	58	33	42	**40**
Agency 8	17	42	25	25	**27**
Agency 9	67	67	42	67	**61**
Agency 10	25	58	83	50	**54**
Agency 11	67	92	92	83	**84**
Agency 12	67	25	17	17	**32**
Agency 13	58	42	67	75	**61**
Agency 14	25	17	8	42	**23**
Agency 15	67	42	58	58	**56**
Agency 16	58	83	75	50	**67**
Agency 17	50	67	50	58	**56**
Agency 18	50	42	42	42	**44**
Total	44	55	49	49	**49**

**Table 3:** Crosstabulations—Concept of Operations and Coordination mechanism of different agencies working in health and other than health sectors during COVID-19 response in Pakistan.

Variables	Functions	Yes	Partially	No
Does your agency has legislative support for national planning and response to COVID-19?	Health	2 (6%)	19 (59%)	11 (35%)
	Other than Health	3 (20%)	5 (33%)	7 (47%)
Does your agency has membership in high-level multisectoral committee at national level during COVID-19 response	Health	13 (41%)	7 (22%)	12 (37%)
	Other than Health	11 (73%)	2 (13%)	2 (14%)
Is there any institutional framework that allows you to promote joint planning and response procedures for health emergencies	Health	0	8 (25%)	24 (75%)
	Other than Health	0	7 (47%)	8 (53%)
Does your agency has a maintained database of staff trained in emergency response and coordination?	Health	0	8 (25%)	24 (75%)
	Other than Health	4 (27%)	2 (13%)	9 (60%)

### Leadership, finance and legislation

**Figure 2:**
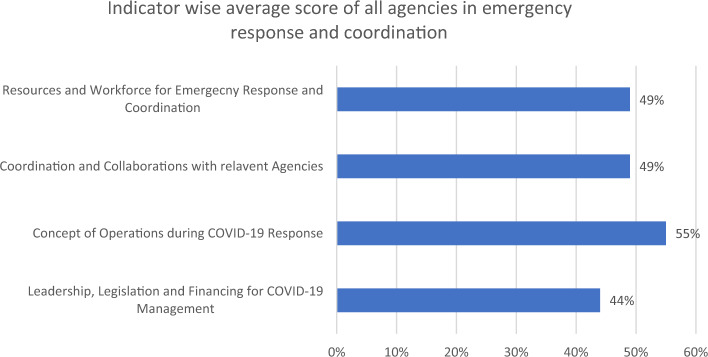
Indicator wise average score of all agencies in emergency response coordination in Pakistan during COVID19 Response.

During pandemic response, all of the organizations documented limited legislative and financial support (44%) for COVID-19 management (Fig. 2). In addition, most of the agencies were scored less in legislative and financial support as compared to other core capacities (Table 2). Sectoral and integrated planning to public health emergency with financial support was also a big issue at the time COVID-19 response (Table 3).

### Concept of operations

**Figure 3:**
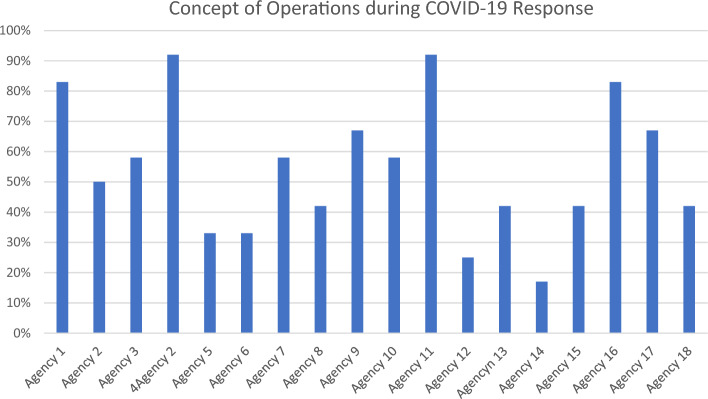
Agency wise average score of Concept of Operations (CONOPs) during COVID-19 response in Pakistan.

Regarding concept of operations (CONOPs), data reveals that the average score in this indicator was 55% as whole (Fig. 2). It reveals that roles and responsibilities attached with designated organizations was relatively clear for most of the agencies. Half of the agencies demonstrated clarity in their roles during response to COVID-19 (Fig. 3). The partner organizations were somehow clear at time the time of pandemic (Fig. 1). Two out of three developmental partners were clearer about their roles and responsibilities during pandemic response (Table 2).

### Coordination and collaborations

Coordination and collaboration with internal, external developmental partners and subnational level entities were big challenges. Coordination and collaboration indicator score ranges from 8–92%. It reveals that some of the organizations achieved very good coordination and collaboration during COVID-19 response on need basis. Whereas none of the agency have fully developed exiting institutional framework that promotes the joint planning and response procedures for public health emergencies (Table 3). From the partners, only one out of three has relatively good score (75%) in coordination and collaboration indicator (Table 2).

### Human resources and workforce

Out of 18 agencies, 10 agencies were lacking (≤ 50%) in skilled and trained human resource in public health emergency response (Table 1, Fig. 2). It was found that none of the health sector agency has mapped the trained workforce, logistic resources, and their deployment prior to COVID-19 emergency. The agencies other than health (non-health sectors) have more resources in relation to health sector, for emergency response coordination (Table 3). The developmental partners and a few organizations have adequate resources in their areas of functions to respond emergency.

## Discussion

Before COVID-19, Pakistan has not formal existing mechanism of multisectoral public health emergency response coordination. Most of the agencies engaged in the pandemic response, faced legal, financial, administrative challenges in developing and maintaining operational support for the emergency. Existing record within the agencies, suggest that an informal and ad-hoc inter-agency coordination strategies were established during COVID-19 response which extended during whole period. Interagency coordination system was backed by the strategic heads of government but not on the basis of on agreements or understandings. Findings reveal some of the agencies and departments were engaged in multiple tasks whereas a few were not clear in their roles and concepts of operations during COVID-19 response. Most of the emergency response coordination arrangements were made on need basis which supported response objectives at national and intermediate level.

It was found that since inception of pandemic Pakistan started emergency response coordination by engaging some relevant sectors especially Point of Entries (POEs), NDMA, NEOC, Central Health Establishment (CHE), WHO country office, UKHSA, CDC and other partners^18^. As the incident escalated the PHEOC expanded coordination and collaboration with all relevant agencies in both, health and other than health sectors. The developmental partners including WHO, UKHSA, JSI, UNICEF and many others supported government agencies in maintaining coordination, workforce capacity building and tactical operations during pandemic response.

The study results highlighted that almost all of the organizations faced challenges in developing their routine capacities in emergency response coordination. Main problem was with legislative and financial support (Fig. 2) in the implementation of activities. Studies have demonstrated that financial and logistics challenges have also been observed for many of the departments and sectors engaged in COIVD-19 response^19,20^. Most of the organizations were not having dedicated funds to support day to day COVID-19 response activities (including testing, surge staff and deployment, quarantine, door to door vaccination etc.)^21^. For immediate nature of tasks, NCOC has coordinated with national and international developmental partners for logistic support in surveillance, medical countermeasure and lab services^12^. The World Bank’s PPR Tool assesses pandemic preparedness in various countries, revealing similar financial and logistics gaps in areas like surveillance, laboratory capacity, and risk communication in Kenya, Cameron and Nigeria^22^.

NIH took the initiative of planning for COVID-19 and developed the National Action Plan for COVID-19 by coordination with stakeholders in March 2020^23^. Although plan including various activities of prevention, detection and response but not facilitate the partner organization’s roles, involvement level, financial regulation, and resources utilization with a clear concept of operation^24^. In May 2020, MoNHSR&C developed a draft of the Pakistan Preparedness and Response Plan for COVID-19, including funding details^12^. It aimed to strengthen disease surveillance, detection, case management, risk communication, infection prevention, and control and provided the ways to reduce coordination gaps. NDMA also developed a Stakeholder Engagement Plan (SEP) under Pandemic Response Effectiveness in Pakistan in May 2020^25^. The plans developed by the agencies facilitated pandemic response but could not fulfill all requirements emergency response coordination^26,27^. Results of present study also reveal that most of the agencies in health sector, faced challenges in developing agreements, activities and timeline, and a clear concept of operations for COVID-19 emergency.

Most of the organizations engaged in pandemic management have different functions and strengths. Prior to COVID-19, the concept of operations and level of engagement for other branches/departments of government organizations and other partners was not clear in Pakistan^28^. Evidence revealed that COVID-19 related planning and operations were being supported by the different ministries and departments including, animal health, law enforcement agencies, information and broadcast, agriculture, civil administration and finance etc.^25^.

The roles and responsibilities were assigned on need basis from the strategic level authorities. There was no clear framework of actions and task assigned to each of the response organization notified in the NAP^23^. Only few of the organizations have defined emergency response activities, parallel to normal day-to-day business.

None of the agencies had endorsed the concept of operations plan or level of engagement for other than health agencies. Evidence from the present study (Table 2) demonstrates that it was unclear how different agencies engaged its branches in the absence of a shared comprehensive response plan. Thus, the unavailability of the documented concept of operations affected the overall planning and emergency response operations collectively.

Prior pandemic, health agencies were deficient in existing coordination for emergency response in Pakistan^29^. At the strategic level, the government of Pakistan constituted a high-level National Coordination Committee chaired by the Prime Minister of Pakistan^24,30^. The aim was to enhance coordination of information and actions required by all national and provincial level agencies^31,31^. Later, NCOC developed effective coordination mechanism among all partners in addition to the COVID pandemic response. Although many agencies involved in coordinated response to COVID, but there was very little evidence on joint planning for the emergency response. During desk review it was found that MOUs with the different departments and partners were not in place at national level.

During the COVID-19 pandemic, Pakistan faced challenges in the field of technical professionals and logistics to manage the emergency^33^. Data show that most of agencies expect developmental partners have a lack of workforce and technical resources for response and coordination (Fig. 1). Literature and departmental record reveal that NCOC is also lacking in permanent trained human resources support to perform all its functions.

Inter-agency human resources exchange was also a big challenge. Pakistan’s Joint External Evaluation (JEE) indicates that Pakistan lags behind in strategic emergency planning, preparedness, resource identification, and mapping^34^. NIH facilitated in capacity building of provincial and regional health departments to generate trained human resources for the emergency response coordination in 2020 and 2021^35^. But the national level response organizations were lacking in capacity for their human resources to be engaged in emergency response at time of escalation. Most of the agencies did not deploy permanent liaison staff at operations center on regularly basis but called for specific assignments.

## Conclusion

Regardless of challenges, Pakistan gradually improved emergency response coordination system through public health emergency operations center platform. The coordination system was on ad hoc basis, lacking in MOUs between different agencies involved in particular response. Some of the agencies achieved a good level of coordination and collaboration for ongoing responses. Due to centralized incident management system, near half of agencies were clear about their roles and responsibilities in pandemic response. Joint planning and response system to deal with emergencies was not properly established. Most of the organizations did not have maintained human and logistics resources mapping and tracking system which is essential to response measures.

Public health emergency preparedness and response are continuous cycles. Legislative, financial, and leadership support with an established concept of operations could improve the preparedness and response against pandemics. Regular resources mapping and establishment of a resources utilization system can improve the public health emergency management system. The public health emergency management system must be able to respond effectively with joint coordination and collaboration form a central point.

### Strengths and limitations

- Assessment of the coordination system of public health emergency operations center (PHEOC) to manage the COVID-19 pandemic by involving different sectors.

- Highlighted how health and other than health (non-health) sectors collectively worked to strengthen the response against COVID-19 in Pakistan.

- The study could not cover all of the response organizations at provincial level that are connected to national level institution/organization for pandemic response.

- All of planning, preparedness and response indicators of public health emergency management were not assess due to time and resource contains.

### Limitations

The study focused on the assessment of coordination mechanism at national level only, role of provincial departments and agencies were out of scope of this study. Due to some administrative sensitivities of certain agencies some strategic, supportive documents, information and financial information could not be assessed. Moreover a few of the agencies listed in the NAP did not provided response as they could not give the consent to provide information.

## Data Availability

The primary datasets used and analyzed during the current study available on request. No human data is used in this study.
